# Supplementary material for: Empirical methods for controlling false positives and estimating confidence in ChIP-Seq peaks
Source: BMC Bioinformatics. 2008 Dec 5;9:523. doi: 10.1186/1471-2105-9-523 (PMC2628906; doi:10.1186/1471-2105-9-523)
Supplement: Additional File 1 — A variety of html documents from the USeq web site detailing the available applications, their best usage, output file type descriptions, command line menus, etc. [file 1471-2105-9-523-S1.zip › USeqUserGuides/JavaDoc.zip!/JavaDoc/trans/tpmap/package-summary.html]

trans.tpmap


|  |  |  |
| --- | --- | --- |
| **Overview** \| **Package** \| Class \| **Use** \| **Tree** \| **Deprecated** \| **Index** \| **Help** | |  |
| **PREV PACKAGE**   **NEXT PACKAGE** | **FRAMES**    **NO FRAMES**     **All Classes** |  |


---

## Package trans.tpmap

For maping, filtering, and calculating statistics about text version bpmap files.

**See:**
  
          **Description**

| **Class Summary** | |
| --- | --- |
| **BlastFilterResult** | Container for a blast result: chromosome, numMatches, exactMatches, oneBPMismatches, startExactMatch. |
| **CollapseTPMap** | Collapses a text version bpmap into a unique set of bpmap lines base on the oligo sequence. |
| **ConvertTPMapsToFeatures** | Converts TPMaps to MapFeature[] and saves them to disk. |
| **FilterTPMapByRegions** | Filters a TPMap file by regions (tab delimited: chr start stop). |
| **IntensityFeature** | Minimal info about a feature for combining multiple chips together. |
| **MapFeature** | Tpmap line broken into some components, chromosome, start, pmX, pmY, and optionally, mmX, mmY. |
| **MapSplitter** | Splits a '.bpmap' text file into different chromosomes, helper script for `TPMapProcessor`. |
| **MummerMapper** | Application to map 1lq files to fasta files. |
| **OligoFlipper** | This reversed the oligo sequence and orientation in a tpmap file. |
| **Stat1lq** |  |
| **StatTPMap** | Calculates simple stats on a tpmap file, number unique oligos, number of lines, gc, tm... |
| **StatTPMapControlSeqs** |  |
| **TPMapDensityReducer** | Randomly drops a certain fraction of tpmap lines. |
| **TPMapDuplicateFilter** | For extracting duplicate oligo/ features from Affy's bpmap file, can be used as a stand alone but better to use `TPMapProcessor`. |
| **TPMapFeature** | Container for info about a particular oligo feature from a tpmap file. |
| **TPMapLine** | Minimal tpmap line, chrom, start, nothing else. |
| **TPMapOligoBlastFilter** | Application for remapping and filtering a text version bpmap file with BLAST. |
| **TPMapProcessor** | App for processing the tpmap file for use by downstream TiMAT applications. |
| **TPMapSort** | This sorts a tpmap first by chromosome, second by base position. |
| **TPMapStripHighLowGC** | Strips a tpmap file of lines where the %GC is > 0.6 or < 0.4. |
| **WindowMaker** | Creates Windows (start position, end position) of a given length, containing a given number of start positions and maximum number of features. |

## Package trans.tpmap Description

For maping, filtering, and calculating statistics about text version bpmap files.
Use the ProbeExporter application from Affy to convert your binary bpmap to a text version.

---


|  |  |  |
| --- | --- | --- |
| **Overview** \| **Package** \| Class \| **Use** \| **Tree** \| **Deprecated** \| **Index** \| **Help** | |  |
| **PREV PACKAGE**   **NEXT PACKAGE** | **FRAMES**    **NO FRAMES**     **All Classes** |  |


---
